# Editorial: Natural remedies repositioned: advancing drug discovery for infectious diseases

**DOI:** 10.3389/fphar.2025.1655921

**Published:** 2025-07-24

**Authors:** John Ogbaji Igoli, Fidele Ntie-Kang, Godwin Unekwuojo Ebiloma, Norzila Ismail, Bamidele Joseph Okoli

**Affiliations:** ^1^Department of Chemistry, Joseph Sarwuan Tarka University, Makurdi, Nigeria; ^2^Center for Drug Discovery, University of Buea, Buea, Cameroon; ^3^School of Science, Engineering & Environment, University of Salford, Manchester, United Kingdom; ^4^ Department of Pharmacology, School of Medical Sciences, Universiti Sains Malaysia, Pulau Pinang, Kelantan, Malaysia; ^5^Department of Chemical Sciences, Bingham University, Karu, Nasarawa, Nigeria

**Keywords:** medicinal plants, drug discovery, antiviral, antifungal, immunomodulatory, COVID-19, traditional medicine

## 1 Introduction

Historically, medicinal practices relied on natural remedies as therapeutic agents. Several drugs and drug scaffolds in use today can be traced to natural products isolated from plants, animals, or microorganisms. However, the systematic and funded study of natural compounds for drug discovery declined with the rise of synthetic chemistry. Today, there is renewed momentum as research seeks to unlock the latent potential of natural remedies and supplements for the development of new therapeutics. The advent of technologies such as high-throughput screening and genomic analysis now facilitates the identification and optimization of bioactive compounds while reducing reliance on synthetic chemistry. The present Research Topic intends to collate manuscripts reporting or describing the identification, isolation, characterization, and preclinical/clinical evaluation of bioactive compounds from natural sources, as well as the exploration of traditional medicine for modern drug discovery. The overall goal is to harness the potential of natural products for developing novel treatments for infectious diseases by consolidating research in this area.

The submitted articles covered three broad areas: antiviral, antifungal, and immunomodulatory potentials of natural products.

## 2 Antiviral properties of natural products

Chihomvu et al. (2024) report on the repositioning of biologically active compounds found in Fuzheng Jiedu (FZJD) granules for a significant reduction in the risk of COVID-19 progression (severe illness, ventilation, ICU) in high-risk patients, especially those in vulnerable groups. Some bioactive compounds were identified, and their mechanisms of action were found to include cytokine suppression, NLRP3 inhibition, metabolism modulation, and lung barrier enhancement. The potential of such compounds provides a dual advantage: they offer a rich source of lead candidates for new drugs and present lower toxicity profiles than their synthetic counterparts.

Another study showed that natural remedies can serve as scaffolds or templates for synthetic modifications, a strategy that can lead to the development of novel agents. Eicosapentaenoic Acid (EPA), a well-known omega-3 polyunsaturated fatty acid abundant in fish oil and breast milk, was found to act as a broad-spectrum antiviral compound by physically disrupting viral envelopes, preventing cell entry, and was observed to be effective against Zika (IC_50_ ∼0.42 µM), Dengue, HSV-1, H1N1 with low toxicity which would enable repositioning. Artemisinin, derived from *Artemisia annua* (Sweet wormwood plant), has been redefined in modern medicine as an effective treatment for malaria (Wang et al., 2019). Through understanding its mechanism of action, researchers are exploring analogs to enhance efficacy or broaden its scope against other infectious agents (Vaou et al., 2021).

## 3 Antifungal effects of natural products

One of the reports highlights the rejuvenation of natural remedies as antifungal agents within the drug discovery pipeline. This aligns harmoniously with the concept of “rediscovery,” where ancient knowledge constructively interferes with modern activity validation. Computational screening identified the marine compounds Naseseazine C and Wailupemycin H, which potently inhibit Yck2 in drug-resistant *Candida* albicans. Simulations confirmed stable binding and superior affinity (lower binding energies: –81.67/–67.12 kcal/mol). The growing body of research lending credence to long-held beliefs about natural products can foster a paradigm shift in how scientists approach drug discovery and development. Collaborative efforts between ethnobotanists and natural products chemists or scientists, synthetic chemists, pharmacologists, and those researching Drug Metabolism and Pharmacokinetics (DMPK) are crucial in this transformation (McClatchey et al., 2009). SAR studies on the monoterpenes, geraniol, citronellal, and linalool revealed that geraniol showed superior antifungal activity (MIC 1.25–5 mM), antibiofilm effects, and lower cytotoxicity against *C. albicans*. Geraniol also downregulated virulence factors (PLB1, SAP1) and suppressed pro-inflammatory cytokines (IL-1β, IL-6, IL-18), repositioning it as a multifunctional antifungal candidate. Nevertheless, the report highlights the challenges of standardization and quality control of natural remedies, a sentiment also highlighted by Ungogo et al. (2020).

## 4 Natural products with immunomodulatory effects

In addition to their intrinsic antimicrobial activities, several natural remedies possess immunomodulatory effects, which can be crucial in combating infections. A pertinent example is echinacea, which is often used for its ability to enhance immune function (Zhai et al., 2007). A meta-analysis found that echinacea significantly reduced the likelihood of contracting respiratory infections and may decrease their duration (Karsch-Völk et al., 2014). By leveraging the immune-boosting properties of these natural compounds, researchers are considering integrative approaches that enhance traditional treatments and vaccination strategies.

Unlike synthetic drugs, which are produced with consistent chemical composition, the efficacy of botanical products can vary widely based on their source, preparation, and dosage. Regulatory frameworks will need to adapt to ensure that natural compounds are rigorously tested and validated for clinical use.

The repositioning of natural remedies signifies a vital resurgence in their exploitation and exploration in drug discovery. Driven by modern science validation and assays, traditional remedies and traditional knowledge can be properly evaluated. Compelling evidence—from FZJD granules (COVID-19) and EPA’s antiviral envelope disruption to marine Yck2 inhibitors and geraniol’s multifunctional antifungal action—demonstrates nature’s potent and diverse molecules. These offer novel mechanisms and lower toxicity. While advanced technologies unlock this potential, standardization challenges require rigorous validation. Ultimately, this synergy between ancient knowledge and modern science provides transformative, patient-centred approaches to addressing evolving global health threats.
